# Impact of supervisors' research style on young biomedical scientists' capacity development as measured by REDi, a novel index of crossdisciplinarity

**DOI:** 10.3389/frma.2022.990921

**Published:** 2022-09-20

**Authors:** Akiko Hashiguchi, Hiroka Hamada, Satoru Takahashi, Keisuke Honda

**Affiliations:** ^1^Faculty of Medicine, University of Tsukuba, Tsukuba, Japan; ^2^Planning Unit, Administration Planning and Coordination Section, The Institute of Statistical Mathematics, Tachikawa, Japan

**Keywords:** research activities, correlation analysis, diachronic effect, collaborative networks, funding, Research Diversity Index (REDi)

## Abstract

The challenge for medical schools in Japan is to develop research activities for innovation. This study aimed at analyzing the connection between the research output of “promising researchers” (next-generation leaders in terms of research activity) and their supervisors' past research activities to identify those factors that impact researchers' performance. Activity was analyzed from the viewpoints of productivity, coauthorship networks, and research impact using a novel index called the Research Diversity Index (REDi) that quantifies crossdisciplinarity. Research funding, which has not yet been fully utilized in correlation studies of the characteristics of authors, was also considered in this study. For the promising researchers extracted using betweenness centrality scores within coauthorship networks, there were diachronic correlations between the records of the promising researchers and those of their supervisors. Supervisor leadership as measured by the number of last-authored publications and extent of networking had a positive effect on the promising researchers productivity. Supervisors' research style of integrating knowledge from multiple fields, as measured by REDi, was negatively correlated with the publication impact of promising researchers, suggesting that REDi is useful as a novel indicator of research quality not being captured by existing indices. It was also noted that establishing an academic presence through extensive collaborations could be advantageous for obtaining research funding, especially from top-down government programs. The possible implications of this study for promoting research activities are the importance of incorporating new doctorates into research groups at an early stage and that of promoting interinstitutional, crossdisciplinary collaborations.

**Classification code:**

MSC: 62P10 Applications of statistics to biology and medical sciences; meta-analysis.

JEL: Z1Z10 Cultural Economics • Economic Sociology • Economic Anthropology- General.

## Introduction

The field of biomedical science has grown significantly as motivated young people participate in research in increasingly large numbers parallel with the expansion of the pharmaceutical and biotechnology sector (Alberts et al., 2014). The rise of biomedical industries through the practical application of research results has been supported by trained research physicians who can lead clinical research based on disease-oriented perspectives (Kozu, 1997; Onishi and Yoshida, 2004; Yin et al., 2015). However, there is a shortage of research physicians although over production of physicians and PhD scientists (Headquarters for healthcare policy, 2014; Yin et al., 2015; Ommering et al., 2021). A decrease in the number of research physicians has been a critical issue for Japan's biomedical research and practice. Due to the 2004 reform of the clinical training system for physicians that mandated two or more years of clinical training, the number of residents remaining in university hospitals has fallen, and graduate students training to become clinicians are no longer choosing to conduct basic research (Sato and Koyama, 2010; Onishi, 2018). As a result, it is becoming increasingly challenging to recruit professionals who have a medical license and conduct basic medical research as professors and associate professors.

In Japan, research activities at national universities are mainly supported by public funds. The government's allocation of funds to national universities is based on the so-called “dual support system,” which consists of covering basic expenses and also offering competitive funds that are allocated according to various policy objectives (Ministry of Education, 2006). One of the major changes in Japanese higher education occurred in 2004, when national universities were incorporated. Since then, funding principles have shifted from general allocation to project-type funding (Ministry of Education, 2006). Escalating demands for accountability of public funding allocation often lead to the evaluation of university performance (Geuna and Martin, 2003; Auranen and Nieminen, 2010). Starting in 2019, a new system of performance-based allocation was introduced, which utilizes indices such as the number of research publications, the amount of external funds obtained, and the impact of publications (Ministry of Education, 2021). Approximately 70 billion yen in FY2019 and 85 billion yen in FY2020 were intended to be allocated through this new system.

Under these circumstances, each university needs high-performing personnel in research activities, and the development of early-career faculty members to conduct research has become an urgent issue. A useful reference for the development of researchers at each university is how highly promising researchers with excellent research capabilities, who are considered to be leaders of the next generation, have been trained. These promising researchers range from those up to about 45 years old, who has the potential to become principal investigators in the next professorial election, to star scientists in their 30s with outstanding achievements. Many studies on factors affecting researcher development have focused on graduate education. Past studies have revealed that supervising professor's support positively affects the publication performance of students during doctoral training and up to 1.5 years post-degree (Shibayama and Kobayashi, 2017; Shen and Jiang, 2021). On the other hand, there is little research on the factors that determine research performance from degree completion to becoming a mid-career professional researcher, and not fully understood except the importance of the current work environment (Way et al., 2019).

Recently, biomedical research has become increasingly resource- and labor-intensive in order to obtain truly high-impact findings, and the importance of team-based research activities has increased (Stephan, 2012). Research groups of medical schools in Japan have a characteristic team structure involving a directing professor, other faculty members, physicians, Ph.D. students, residents, and in some cases, physicians of affiliated hospitals; each of these members will be assigned a specific research task based on their professional situation (Ikai, 2000). It is not surprising that the productivity of the team members is significantly influenced by that of the directing professors since achievements during the same period are published as coauthored papers with the directing professors as supervisors. To identify good supervisors who will develop their team members into high-performing researchers and the factors that affect the training process and outcomes, it is important to clarify the correlation between the current activity of the team members and the past scientific contributions of the supervisors. Another reason to focus on the diachronic correlation is that the research paths that supervisors have taken may be a reference for the project team members, particularly in the case of mid-career academics who are on the verge of becoming next-generation leaders. Yoshikane et al. (2009) have suggested that supervisors' networking styles, i.e., whether they act as leaders or followers in their collaborative networks, affect the future publication performance of their team members.

In this study, we analyzed the diachronic correlation between the research activities of promising researchers considered to be next-generation leaders at each university and their supervisors as defined as the most frequent coauthors. Activity was analyzed from the viewpoints of productivity, coauthorship networks, funding, and research impact. To measure the crossdisciplinarity of publications, a novel index called the Research Diversity Index (REDi) was developed for the first time. Research evaluations often employ indices that express the level of attention in a deeply specialized and segmented field in historical academic systems. However, the fragmentation of disciplines makes it difficult for each research domain to grasp and address today's socioeconomic issues involving numerous factors. Not only that, it discourages the free generation of the ideas that become new knowledge and turns research into a competition in which the researcher only performs conventional movements within a defined field. It became necessary to identify and promote efforts where multiple disciplines are collaborating to create new fusion fields. Here, we propose REDi as an index that considers the distance between the fields in which an article has been cited; specifically, articles cited in more distant fields have higher REDi values and thus the index indicates the breadth, not the depth, of a study's impact. This index was used to describe a researcher's research style, taking due account of application-oriented nature of biomedical research, which includes research with a strong practical aspect such as medical device development.

## Methods

### Samples and data source

The paucity of studies on the performance of researchers up to the mid-career level in Japanese medical schools may be related to the fact that most of them do not aim to become research physicians immediately after obtaining their medical licenses, but instead take a faculty position as a clinician that does not have research as its primary responsibility, which makes it difficult to identify the starting point of their academic careers. This study focuses on a single university in order to obtain detailed microstructural data to track the careers of researchers. We selected the Faculty of Medicine at the University of Tsukuba as the target institution because the University of Tsukuba is a research university and a member of the Designated National University Corporations that are expected to significantly improve their education and research standards while at the same time playing a key role in local medical care as running an advanced treatment hospital as defined by the Medical Care Act.

We used a Neo4j-based graph database, called ISM+Neo4j (The Institute of Statistical Mathematics, Japan), that consisted of 30 years of Web of Science data on articles published until 2016 provided by Clarivate Analytics for measuring the productivity of researchers, coauthorships, and citation networks. To measure researcher productivity, research indices related to coauthorship and contributions as expressed by author rank were calculated. We proceeded with the analysis assuming that the first authorship position represents largest workload and the last authorship position represents leadership based on Shulkin et al. (1993) and Bhandari et al. (2014) where it was indicated the last author is considered the second most prestigious position after first author and is the supervisor. The authorship as last author was confirmed from the reprints of the papers. Research impact was quantified using Source-normalized Impact per Paper (SNIP) scores from the SciVal database (Elsevier, Netherlands) under license by the University of Tsukuba. The external research funds obtained were extracted from the Database of Grants-in-Aid for Scientific Research (National Institute of Informatics, Japan); information on other government funding was retrieved from a database covering most of the government funding programs (https://research-er.jp/).

### Overview of Research Diversity Index (REDi)

**Figure 1:**
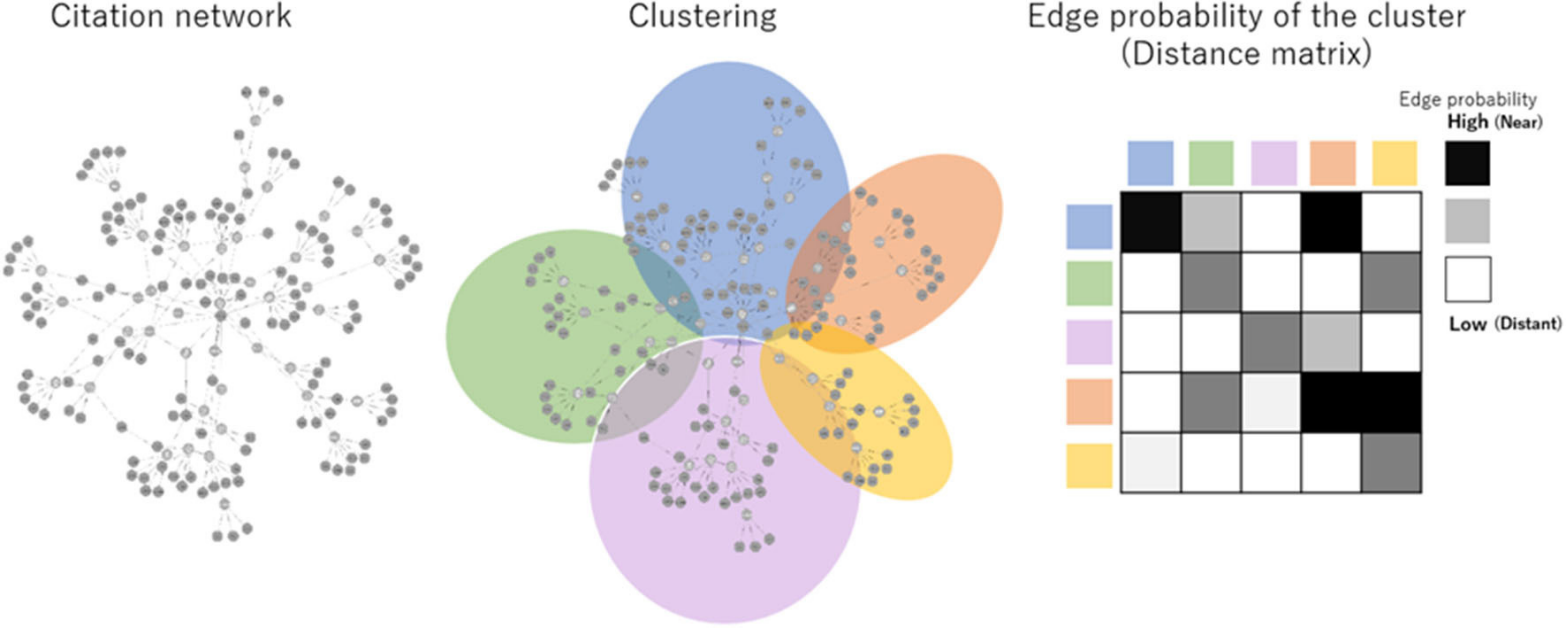
Basic idea of REDi. Whole citation network, in which nodes represent papers and edges represent citations, respectively, is clustered to redefine fields. The distance between fields is obtained as the likelihood of citation occurring between clusters using a distance matrix generated by SBM.

**Figure 2:**
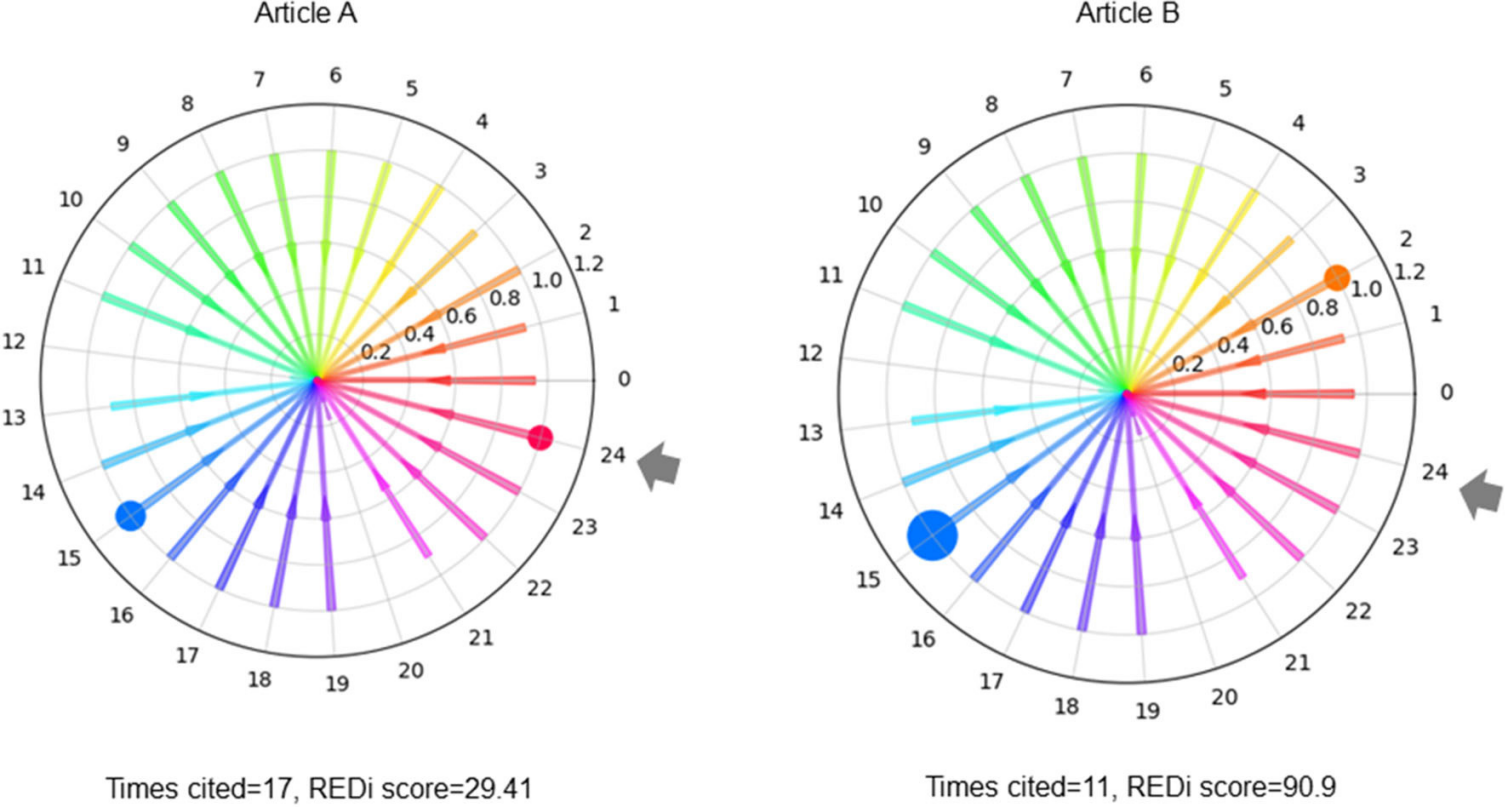
Citation status and REDi score. Even for papers cited to the same extent, the value of REDi will be greater if the paper is cited more frequently from a different field than the field to which it belongs. Numbers indicate fields. Gray arrows indicate the field to which the paper to be evaluated belongs. Bubbles indicate citation frequency in the field.

REDi focuses on the citation-citation relationship of bibliographic data to measure the degree of heterogeneity in academic fields, redefines the fields based on their overall structure, and scores each paper as a distance in terms of the likelihood (probability) of a citation relationship occurring between the fields. The most basic idea to calculate REDi is the fact that papers cite strongly related papers to their own research. There is a study by Mysore et al. (2022) that is based on the same idea. In this study, the degree of relatedness strength is replaced by the distance between the fields. By clustering whole citation network covering all papers, the degree of relatedness strength, i.e., the distance between fields, is obtained as the likelihood of citation occurring between clusters (Figure 1). In addition, it can redefine potential fields that are not based on existing disciplines (Figure 1). Figure 2 shows two papers with similar number of citations but different score of REDi. Even for papers cited to the same extent, the value of REDi will be greater if the paper is cited more frequently from a different field than the field to which it belongs (Figure 2). This is a key feature of REDi, that takes into account actual relationships between papers, which cannot be considered when using pre-defined fields provided by the bibliographic database.

**Figure 3:**
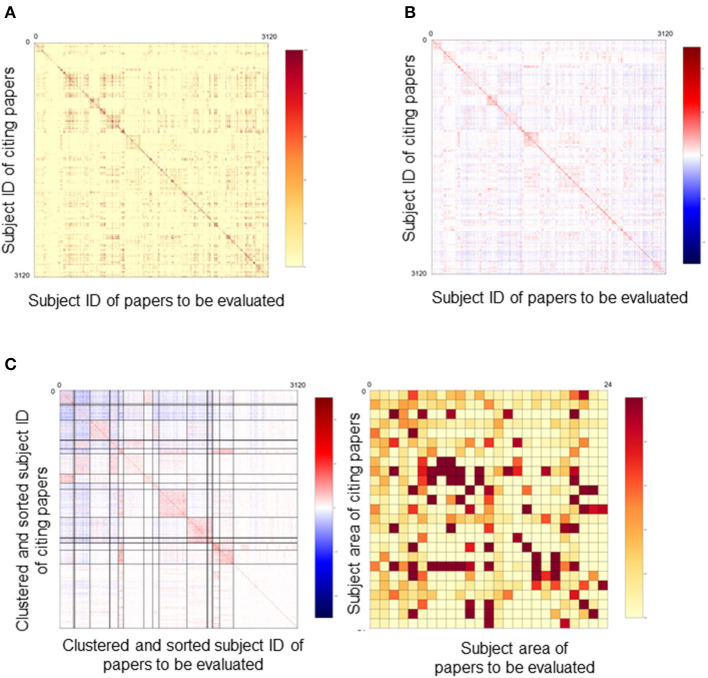
Clustering of papers. **(A)** Adjacency matrix of the citation-citation network. About 3,200 aggregation patterns of class were detected. Each class is assigned a unique subject ID number. The number of citations for each element were shown in a color chart. Areas with a high number of citations are indicated in red. **(B)** Amount of Self Mutual Information (Pointwise Mutual Information), PMI. The adjacency matrix is normalized by PMI. Areas with a high number of citations are indicated in red. **(C)** Results of clustering by SBM. Clustered subject IDs were sorted and expressed as subject area by taking the average of Subject IDs contained in the cluster.

**Figure 4:**
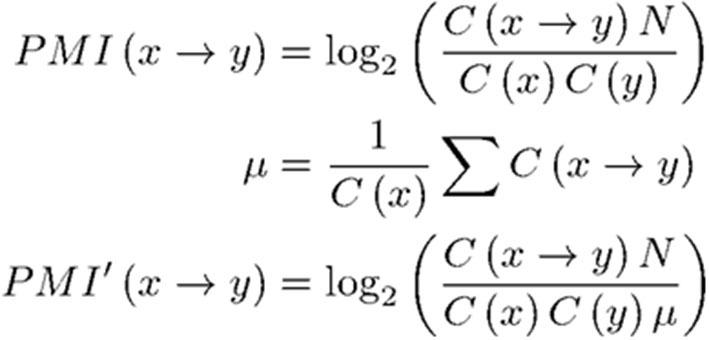
Normalization by PMI.

From here, specific calculations are explained. The score for each paper is calculated by the following steps. First, clustering is performed on data extracted only from citation-citation relationships in the bibliography. The Stochastic Block Model (SBM) (Holland et al., 1983) is used as the clustering algorithm. The data are represented as an adjacency matrix of a directed graph. Since the adjacency matrix of the entire bibliographic data (e.g., 30 years of available Web of Science data) would be very large, the matrix is aggregated in a linear manner. That is, the number of citations between classes of journals is used, where these classes are aggregated by the subject category assigned to the journal (Clarivate Analytics, 2020). There are about 3,200 aggregation patterns of class within the used data. Figure 3A shows the number of citations for each element in a color chart. These aggregation patterns of class are the most basic data in calculating REDi. Each class is assigned a unique subject ID number. This adjacency matrix is clustered using SBM after normalization. This is done because the number of journals covered by each subject ID varies by field. As a normalization method, we use the method of Pointwise Mutual Information (PMI), which is often used in natural language processing to determine the co-occurrence probability of words in a document (Church and Hanks, 1990). The PMI transformation from one Subject ID x to another Subject ID y is expressed as follows, where C (x) is the number of papers in Subject ID x (Figure 4).

C (x → y) is the number of citations from x to y and N is the number of papers in the entire bibliography. This transformation allows us to correct for bias arising from the number of the papers. The matrix after PMI transformation is shown in Figure 3B.

SBM is a kind of probabilistic generative model of graphs. It is based on the assumption that a node belongs to only one class (hard clustering), and that the probability of the existence of an edge from one node to another depends only on the strength of the relationship between the classes. In the case of REDi, a group of about 3,200 Subject IDs has K upper classes depending on the pattern of citation relationships, with the stronger the relationship, the higher the probability that an edge will be created between the two classes (probability of citation). There are several possible algorithms for estimating the K × K probability matrix, and we employed maximum a posteriori probability (MAP) estimation as a hierarchical Bayesian model assuming that.

(a) the probability that a particular cluster contains a node follows a categorical distribution.

(b) the parameters of the categorical distribution follow a Dirichlet distribution (conjugate prior distribution).

The number of clusters, K, is given a priori as a censoring condition for optimization, and is set to 25 in agreement with academic fields based on Essential Science Indicator (Clarivate Analytics, 2020). The resulting probability matrix is represented in Figure 3C. The left part shows the results sorted by subject ID class, with the thick horizontal and vertical lines indicating the boundaries of the clusters. The right matrix shows the probability of connection (edge) merging for each class.

This probability matrix is used to calculate REDi score according to the following procedure.

**Figure 5:**
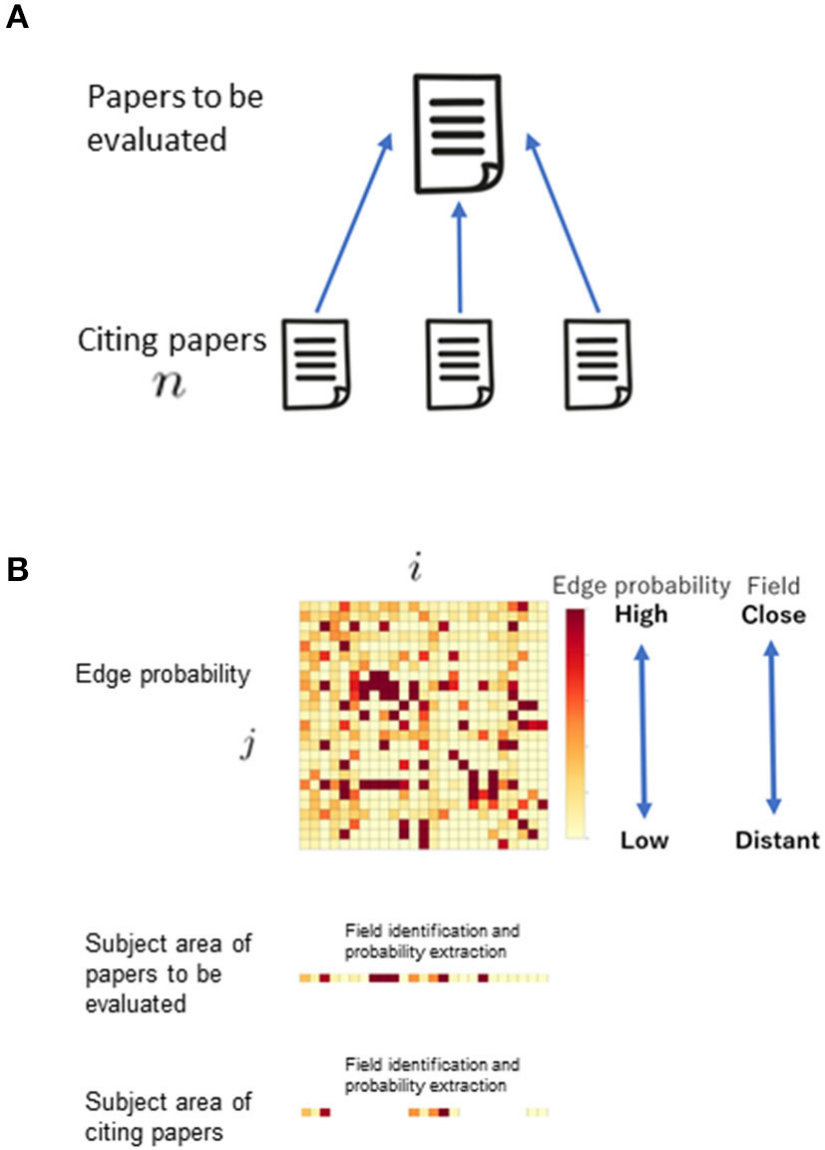
Calculation of distance between fields. **(A)** Papers to be evaluated and papers citing them. **(B)** Acquisition of distance between fields expressed in terms of connection (edge) probability. To calculate REDi score, the fields were identified first, and then the probability for each connection were extracted.

(1) Identification of the fields to which the papers to be evaluated and the papers that cite those papers belong among the predefined 25 fields (Figure 5A).

(2) Calculation of the score using the connection (edge) probability (Figure 5B).

**Figure 6:**
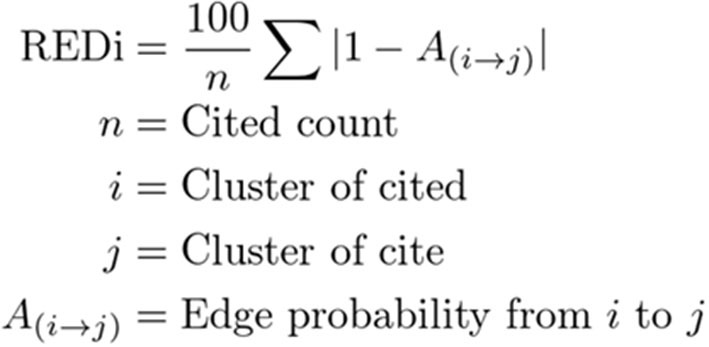
Calculation of the score of the diversity index.

After identifying elements of the matrix of edge probability of SBM results from the pairs of Subject IDs of the papers to be evaluated and that of the papers which cite those papers obtained in (1) (Figure 5B), the average of these values is the value of the diversity index, REDi (Figure 6).

### Analysis

The researchers to be analyzed were those whose betweenness centrality scores in the coauthorship network increased in the 2012–2016 period; the 5-year period is consistent with the time period for evaluation of researchers as seen for many tenure-track positions. The period was set to 2012–2016 because the Web of Science data provided by Clarivate Analytics covered the period until 2016. Accordingly, the period for precedent activity measurement of the supervisors was 2007–2012. As the research environment in universities in Japan underwent significant changes in 2004 with the incorporation of universities, we aimed to minimize the impact of the reform by setting the period after 2007. Although a lag time is expected before publication, by targeting the next-generation leaders as subjects, we observed the continuing activities of those who have conducting their research activities at the University since before 2012. Articles with at least one author affiliated with the Faculty of Medicine, University of Tsukuba were retrieved for 2012–2016 using the search terms “Univ Tsukuba” and “Med” for organization and suborganization categories, respectively; a total of 1,900 publications that met this search criterion were retrieved (Table 1).

**Table 1:** Description of data.

	**2012**	**2013**	**2014**	**2015**	**2016**
Number of articles retrieved from ISM-Neo4j^a^	257	290	476	449	428
Number of authors extracted from ISM-Neo4j^b^	467	606	695	709	671
Number of authors after collation^c^	406	540	602	639	631
Number of enrolled faculty members^d^	421	432	465	486	459

^a^Articles with at least one author affiliated with the Faculty of Medicine, University of Tsukuba were retrieved using the search terms “Univ Tsukuba” and “Med” for organization and suborganization categories, respectively.

^b^Total number of authors in the data.

^c^The number of authors after collation of the person with the same name.

^d^Retrieved from https://www.md.tsukuba.ac.jp/top/activities/. Approximately 10% of these enrolled faculty members were selected as the top tier (40 researchers).

**Figure 7:**
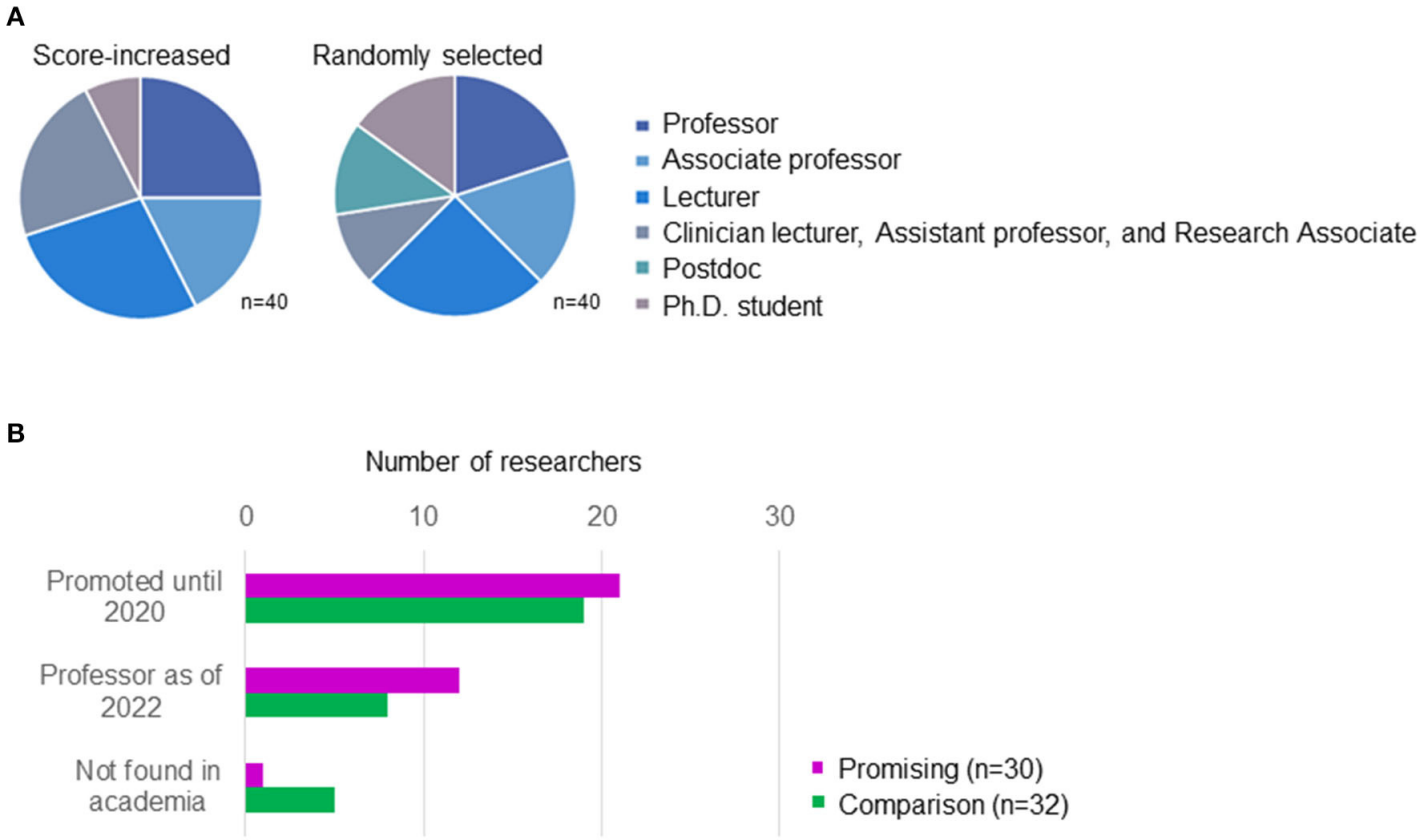
Selected researchers. **(A)** Breakdown of positions of selected researchers as of 2012. Of these, the portion excluding professors is included in the analysis. **(B)** The status of promotion of researchers subjected to analysis.

We employed betweenness centrality, which indicates how much a given node is in between others, to detect subjects, i.e., promising researchers considered to be next-generation leaders because it has been used to identify key role researchers in a research institution (Mizukami et al., 2016) and changes in scores over time have been observed during the career development of top-notch young researchers in Japan in the analysis using data of research fellows of the Japan Society for the Promotion of Science (Fujita et al., 2019). Annual betweenness centrality for each researcher was calculated using Cytoscape (ver. 3.4.0). The researchers were ranked according to the increase in score from the 2012–2013 average to that of 2015–2016, and the increasing trend was confirmed by regression slope. The top 40 researchers, accounting for approximately 10% of all enrolled faculty members during the period, excluding those not engaged primarily in research (Table 1), were selected. We then excluded those who were professors as of 2012 and remaining 30 are defined as “promising researchers” who are considered to be next-generation leaders (Figure 7A). For comparison, 40 researchers not selected in the previous step were randomly selected, and 32 researchers except professors as of 2012 were used as the comparison group (Figure 7A). The distribution of positions tended to be higher for those identified as having increasing betweenness centrality scores. The status of promotions until 2022 indicated that promising researchers were promoted faster and in greater numbers (Figure 7B), and the method is considered reasonable as a method for identifying the next-generation leaders.

The correlation between supervisors' past activities and the research activities of the promising researchers was analyzed as follows: first, the most frequently observed coauthor of each promising researchers was identified during 2012–2016, and the information on articles published by those coauthors within the 2007–2011 period was then obtained from ISM-Neo4j using the supervisors' names as search terms for the author category. The search term for the organization category was “Univ Tsukuba.” For those supervisors employed at other universities during this period, the names of those institutions were also used as the search term. The indices used to measure productivity were the total number of articles published, and the numbers of articles published as first author, second author, and as last author. The number of coauthors and the percentage of coauthors belonging to different institutions of target researchers were employed as indicators of degree of networking. Research impact was quantified for each person using citation count-based indices, namely REDi scores and SNIP scores, averaged per publication. Competitiveness in obtaining research funding was measured by the number and amount of Grants-in-Aid projects and other government research projects for the principal investigator.

The Spearman's rank correlation coefficient (Rs) between the research activities of promising researchers and the past scientific contributions of their supervisors was calculated using XLSTAT software (Addinsoft, France). To identify factors that distinguish emerging young researchers from other academics, principal component analysis (PCA) was conducted using the data of researchers mentored by high-performing supervisors, from both the promising and comparison groups, after correcting their research activities for their supervisors' research activities. In addition, we tracked the acquisition of external funds longitudinally until 2021.

## Results

The definition of “high-performing” research personnel varies depending on the purpose of the analysis. To confirm the validity of the selection of promising researchers considered to be next-generation leaders in our analysis sample, we measured research activity using three indices considered important to enter an academic career. As shown in Table 2, individuals identified as promising researchers had published an average of 4.6 more papers than did the controls from 2012 to 2016. In terms of funding, the promising researchers had an average of 0.57 more Grants-in-Aid projects allocated to them, suggesting that increase in betweenness centrality score can be used as an indicator of active personnel (Table 2). Table 3 presents the results of calculating the correlation coefficients between the performance of the 30 promising researchers during 2012–2016 and the preceding performance of their supervisors (2007–2011). Descriptive statistics for the data underlying this calculation are shown in Supplementary Table 1. The absolute values of the correlation coefficients are within 0.3–0.7, suggesting a fairly strong correlation (Table 3).

**Table 2:** Performance of researchers (mean values) during 2012–2016.

	**Promising**	**Comparison**
	(*n* = 30)^a^	(*n* = 32)^a^
Number of articles	15.60*	11.00*
Number of articles published as the first author	1.73	1.75
Number of GIA projects	1.63*	1.06*

^a^The breakdown by positions as of 2012 is as follows: Promising group: Associate professor 7, Lecturer 11, Lecturer (clinicians), Assistant professor, and Research associate 9, Researcher 0, and Ph.D. student 3; Comparison group: Associate professor 7, Lecturer 10, Lecturer (clinicians), Assistant professor, and Research associate 4, Researcher 5, and Ph.D. student 6.

*Significant (p < 0.05).

GIA, Grants-in-Aid.

**Table 3:** Diachronic correlation between the performance of promising researchers and their supervisors.

	**Performance of promising researchers** ^**d**^							
	**Number of articles**	**Number of articles (first author)**	**Number of articles (second author)**	**Number of co-authors**	**Co-authors from different institutions (%)**	**SNIP per publication^c^**	**Number of GIA projects^e^**	**Amount of GIA projects^e^**
**Performance of supervisors** ^**a**^								
**Productivity** ^**a**^								
Number of articles	0.571	0.362		0.422				
Number of articles (first author)						0.407		
Number of articles (last author)	0.626	0.470		0.506				
**Degree of networking** ^**a**^								
Number of co-authors	0.615			0.631				
Co-authors from different institutions (%)	−0.452							0.393
**Impact of research**								
REDi per publication^b^						−0.455		
SNIP per publication^c^	−0.614			−0.421				

Spearman's rank correlation coefficients (α < 0.05).

^a^Based on publication during 2007–2011.

^b^Calculated using citation count until 2016 (ISM+Neo4j).

^c^Calculated using citation count until 2021 (SciVal).

^d^Based on publication and external funding obtained during 2012–2016.

^e^GIA, Grants-in-Aid.

In terms of productivity, the number of the supervisors' articles, as well as that of those published as last author, has a positive effect on the number of promising researchers' articles; a similar relation was observed between supervisor productivity and the number of promising researchers' first-authored articles (Table 3). The number of supervisor coauthorships also exhibited a positive correlation with promising researchers' publication performance (Table 3). The potential reason for this effect is that supervisors who have larger collaborative networks may work on a variety of topics, and as a result, the number of articles is expected to be higher for their team members. The percentage of coauthors affiliated with different institutions had a negative effect, in contrast to the positive effect of the number of coauthors (Table 3); the reason for this outcome needs to be assessed along with the impact of publications (Table 3). A higher SNIP score per publication indicates that supervisors have been publishing, on average, in high-impact journals; presumably, they concentrate their resources, including time, labor, research funds, and laboratory equipment, on achieving high-impact results. The publication policy of these supervisors is “small numbers—high impact,” resulting in a negative correlation between supervisor SNIP scores and their team members' productivity. In addition, it is of crucial importance to have access to the advanced knowledge of top researchers who are often external talent from the home institution through collaborative relationships. If the payoff from research collaborations is significant scientific value expressed in the form of high-impact publications, then fewer papers will be produced collaboratively with coauthors from different institutions.

Regarding the expansion of the promising researchers' collaborative relationships, a positive effect of supervisors' productivity and network size was observed (Table 3). The positive impact of the number of last-authored articles by supervisors is noteworthy because it suggests that supervisors who act as project leaders in their relevant fields attract suitable collaborators to work with their team members. Supervisor SNIP scores had a negative effect on the number of promising researchers' coauthors (Table 3). This may suggest that the most advanced research comes from a small group of people led by an originator of innovative research themes, albeit on different scales. Factors that affect the promising researchers' publication impact as measured by SNIP score per publication include the number of supervisor first-authored articles and the REDi score per publication (Table 3). It is assumed that the supervisors who have published more as first authors have a clearer idea about their research questions; therefore, they are able to motivate their team members to produce high-impact results. Meanwhile, REDi and SNIP are indices that express different perspectives; REDi and SNIP respectively represent breadth and depth of impact. REDi was specifically developed to measure the degree of crossdisciplinarity, which existing indices cannot capture. In this analysis, a negative correlation was observed between supervisor REDi scores and the promising researchers' SNIP score (−0.455) (Table 3), indicating that research style is passed down from supervisors to their team members, whether to pursue cutting-edge research in one field or to integrate knowledge from multiple fields.

Another interesting finding is that the more coauthors a supervisor has from other institutions, the greater the amount of funding from Grants-in-Aid projects a promising researchers receives (Table 3). It is not surprising that the correlation does not appear in the number of projects in a 5-year study period (2011–2016), given that the implementation period of Grants-in-Aid projects is roughly 3 years in most cases, and there are few opportunities to apply for more grants once a project has been selected. The difference in funding amounts is thought to be due to differences between the types of programs from which grants are obtained. Current results imply that promising researchers whose supervisors are well known through direct cooperative relationships are more likely to be selected as recipients in a large funding framework. As active supervisors can boost the productivity of promising researchers (Table 3), the larger financial allocations may be a response to their publication record. However, when the correlation coefficients between each promising researchers' own publication performance and the status of funding are calculated, no clear effect was found for the number of articles or first-authored articles, or SNIP score (Table 4). Instead, the number of second-authored articles had a positive effect on the success in obtaining research funds. Being a second author indicates that the researcher was assigned a particular role in a team and was able to make important contributions. Along with the benefit of working under a renowned supervisor, this result was thought to indicate the significance of gaining recognition as a researcher.

**Table 4:** Synchronic correlation between research performance and funding acquisition of the promising researcher.

	**Grants-in-Aid**	
	**Number of projects**	**Amount of allocation**
Number of articles		
Number of articles (first author)		
Number of articles (second author)	0.417	0.486
Number of co-authors		
Co-authors from different institutions (%)		
SNIP per publication		

Spearman's rank correlation coefficients (α < 0.05).

Based on publication and external funding obtained during 2012–2016.

**Figure 8:**
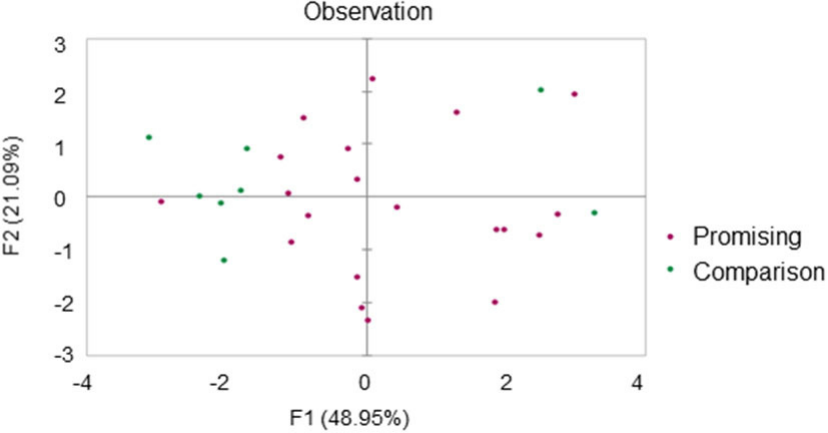
Principal component analysis (PCA) for the researchers' research performance corrected by their supervisors' research performance. Twenty-eight researchers trained under seven prominent supervisors were selected. Purple and green dots indicate the promising and comparison young researchers, respectively.

Not all team members working with high-profile supervisors necessarily become active. To obtain insights into those factors that shape academics into promising researchers, PCA was conducted on the research performance data of researchers supervised by “leading” professors (Figure 8). Seven leading professors were selected based on department size, publication quality, and research funding, and the research performance data of 28 researchers in total, including 20 promising researchers and 8 comparison researchers, were corrected for the research activities of their supervisors. The results showed that the researchers could be divided according to performance: into a group with a large number of individuals in the comparison group with relatively low research activity, and a group containing most of the promising researchers (Figure 8). The latter group was further divided into two groups of either moderate or high research activity (Figure 8). However, the key factors that distinguish the promising researchers were not fully elucidated because the factors were working in combination (Table 5).

**Table 5:** Eigenvectors.

	**F1**	**F2**	**F3**	**F4**	**F5**
Number of articles^a^	0.489	0.000	0.340	−0.269	0.093
Number of articles (first author)^a^	0.197	0.491	0.406	0.731	0.119
Number of articles (second author)^a^	0.321	0.383	−0.448	−0.223	0.686
Number of co-authors^b^	0.439	−0.075	0.496	−0.377	−0.118
Co-authors from different institutions (%)^c^	0.054	0.695	−0.219	−0.256	−0.629
Number of GIA projects^a^	0.467	−0.191	−0.354	0.280	−0.217
Amount of GIA projects^a^	0.454	−0.296	−0.316	0.238	−0.224

^a^Corrected by number of supervisor articles.

GIA, Grants-in-Aid.

^b^Corrected by number of supervisor co-authors.

^c^Corrected by supervisor percentage of co-authors from different institutions.

As an interesting point, the high-activity group contained two comparison group researchers that were not identified as promising researchers based on the change of the betweenness centrality score during the research period. One of these comparison researchers was pursuing original research topics independently on a tenure track and the other was working on applied topics that were not considered mainstream by their research group. This common situation of cooperation outside their own lab being important may have contributed to their high performances, perhaps through collaborations with influential researchers. Table 6 presents the average betweenness centrality score in 2016 for each group within a coauthorship network among collaborators belonging to different institutions. More active groups scored higher, suggesting that becoming visible through direct interaction with other researchers *via* coauthored papers is the key to the development of the young researchers. Finally, we tested the effect of being visible in each academic field on long-term success in obtaining research funding (Table 6). The numbers of projects of Grants-in-Aid and other government funding programs until 2021 were both higher in groups with greater research activity, and the correlation coefficients between the number of projects and the betweenness centrality score with outside coauthors indicated significantly positive effects on other government funding programs (Table 6).

**Table 6:** Visibility in academic fields and competitiveness in funding acquisition.

	**Research activity** ^**c**^			**Coefficients^e^**
	**Low**	**Moderate**	**High**	
	**(*n* = 7)^d^**	**(*n* = 12)^d^**	**(*n* = 9)^d^**	
Betweenness centrality score with co-authors from different institutions^a^	0.004	0.046	0.052	
Number of GIA projects^b^	1.14	2.17*	4.67*	
Number of other government funding projects^b^	0.00	0.33	0.44	0.482

^a^2016.

^b^2012–2021.

^c^Divided into groups based on Figure 1.

GIA, Grants-in-Aid.

^d^The breakdown of groups is as follows: Low: Promising group 1, Comparison group 6; Moderate: Promising group 12, Comparison group 0; High: Promising group 7, Comparison group 2.

*Significantly different compared to low group (p < 0.05).

^e^Spearman's rank correlation coefficients between the betweenness centrality score with co-authors from different institutions (α < 0.05). Calculated using data of twenty-eight researchers.

## Discussion

This study revealed that the increase in the betweenness centrality score within a coauthorship network can be used as an indicator to identify active research personnel and that research activities of promising researchers are influenced in several respects by the past activities of their supervisors, including research style as measured by REDi or SNIP. It is of crucial importance for researchers to be incorporated in the collaborative coauthorship networks of their supervisors, as well as to participate in larger academic networks extending outside their home institution to drive them to conduct further research. The possibility of over-estimation of co-authorships by gift authorship cannot be ruled out with the method used, but Ohata et al. (2020) shows that researchers being hired under a scheme which can foster talented researchers with a high h index, have a higher number of coauthorships than other researchers. Thus, this approach, which uses the betweenness centrality increase along with career development, can be considered reliable in some extent. A separate study of the impact of patterns of authorship and gift authorship is required. Establishing an academic presence through collaborations is advantageous for obtaining future research funding. The present results are based on a small sample of 30 pairs of promising researchers and supervisors, which may present challenges in terms of generalizing the results, but allows, nevertheless, detailed contextual consideration in interpretation, including researchers' personal histories, current roles in their teams, and research interests. The stories interpreted here, which are visualized numerically, are not so different from the impressions of experienced researchers having central roles in their research communities.

In contrast to a previous study using computer science as the target domain that did not find a clear correlation between the productivity of researchers and their supervisors (Yoshikane et al., 2009), the present study found strong correlations in the field of biomedical science. The large number of publications in this field and the strong team system with directing professors at the top may make it easier to see correlations; however, this result is more due to excluding researchers whose betweenness centrality scores did not increase. These researchers may not always be inactive, but sometimes, in contrast, achieve remarkable results even if they are not trained within an established research team, as shown in Figure 8. There were no obvious correlations between research activity of comparison researchers and their supervisors (data not shown), unlike for the sample of promising researchers alone, and the results presented by Yoshikane et al. (2009) would seem to indicate a mixed state of various types of researchers before stratification.

The most important contribution of the supervisors to the development of their team members is including them in a peer group or academic society that shares a common interest in highly specific research subjects. Being integrated into academic societies and networks not only leads to practical benefits, such as an increase in the number of publications or citations, but also produces indirect benefits. Academic societies play indispensable roles in visualizing the distribution of the research population and establishing review categories, the basic unit in grant allocations by field. Being recognized by an academic community through affiliation with research networks helps emerging young researchers obtain Grants-in-Aid in the early stages of their careers and access other government funding programs in the later stages. The reason for this advantage in obtaining grants in these situations is that researcher can refine their research theme to more well-structured research theme to attract the interest of experts through participating in the academic society, as was cleverly put "peer review is a collaboration” in the official magazine of The Oceanography Society of America (Boss, 2018). More technically, professional societies such as the Perinatal Research Society of America helps young investigators obtain NIH funding by offering immersive workshops with one-on-one feedback on writing logical, stimulating, and persuasive applications (Joss-Moore et al., 2022). This would also be an opportunity for young investigators to become known to and to gain the trust of key figures in the academic society. In programs where research themes are predetermined by policy, such as the Plan for Promotion of Medical Research and Development, researchers well known to the screening committee are more likely to be selected and have a greater likelihood of achieving the desired outcome. It is also possible that those who were trained by prominent supervisors influencing national research policy are more likely to be selected because they can craft research proposals that meet the program requirements.

However, the significance of networking is not limited to obtaining funding. Granovetter (1973) analyzed human connections and showed that “weak ties” bring more fresh, unexpected information, and ultimately produce greater results. Research exchanges with many colleagues contribute to the diversification of information sources and the deepening of perspectives, allowing young researchers to select research themes of greater academic interest and social significance (Ohata et al., 2020). Interaction with other researchers may also be useful in fostering young physicians who can lead future clinical research, which requires collaboration among different disciplines and coordination among numerous stakeholders. There are several potential implications of the present study: (i) the usefulness of betweenness centrality scores to identify future high-performing researchers; (ii) the usefulness of REDi to measure the crossdisciplinarity of research; (iii) the importance of incorporating new doctorates as team members into research groups at an early stage; and (iv) the value of promoting interinstitutional collaborations. Information about the factors that led independent researchers to achieve remarkable results remains to be elucidated. A recent report indicated that Nobel Prize-class research topics tend to be published by a small group of people regardless of their past achievements (Ohniwa et al., 2022). Thus, support is needed for new doctorates of two kinds: that to develop them through a team system within a university, and that to nurture those who conduct independent research outside the team system by facilitating interinstitutional, crossdisciplinary cooperation.

## Data availability statement

The data analyzed in this study is subject to the following licenses/restrictions: access to these data is permitted by the ISM Cooperative Research Program. Requests to access these datasets should be directed to AH, hashiguchi.akiko.ge@u.tsukuba.ac.jp.

## Author contributions

AH: conceptualization, data curation, investigation, visualization, writing—original draft preparation, and writing—review and editing. HH: software and data curation. ST: resources and supervision. KH: resources, development of index, and writing—original draft preparation. All authors contributed to the article and approved the submitted version.

## Funding

This work was carried out under the ISM Cooperative Research Program (Grant Nos. 2020-ISMCRP-2016 and 2021-ISMCRP-2019) of The Institute of Statistical Mathematics, Japan to AH.

## Conflict of interest

The authors declare that the research was conducted in the absence of any commercial or financial relationships that could be construed as a potential conflict of interest.

## Publisher's note

All claims expressed in this article are solely those of the authors and do not necessarily represent those of their affiliated organizations, or those of the publisher, the editors and the reviewers. Any product that may be evaluated in this article, or claim that may be made by its manufacturer, is not guaranteed or endorsed by the publisher.

## Abbreviations

REDi: Research Diversity Index
SNIP: source-normalized impact per paper.
