# Supplementary material for: Impact of supervisors' research style on young biomedical scientists' capacity development as measured by REDi, a novel index of crossdisciplinarity
Source: Front Res Metr Anal. 2022 Sep 20;7:990921. doi: 10.3389/frma.2022.990921 (PMC9531751; doi:10.3389/frma.2022.990921)
Supplement: Supplementary file 1 [file Table_1.docx]

Supplemental Table 1. Descriptive statistics of the promising researchers and their supervisors.

|  | Promising researchers  (n=30) | Supervisors  (n=16) ^f^ |
| --- | --- | --- |
| Number of articles ^a^ | 15.6±8.5 | 40.6±24.6 |
| Number of articles (first author) ^a^ | 1.7±1.8 | 1.2±2.3 |
| Number of articles (second author) ^a^ | 2.8±3.6 | 2.3±3.4 |
| Number of articles (last author) ^a^ | - | 22.3±21.5 |
| Number of co-authors ^b^ | 199.3±126.8 | 401.1±223.5 |
| Number of institutions ^b^ | 22.6±14.3 | 40.7±19.8 |
| Co-authors from different institutions (%) ^b^ | 0.3±0.2 | 0.5±0.2 |
| Totaled REDi ^c^ | - | 956.6±444.3 |
| Number of papers which had REDi score ^c^ | - | 26.0±11.7 |
| REDi per publication ^c^ | - | 27.0±8.3 |
| Totaled SNIP ^d^ | 27.2±17.0 | 71.8±33.9 |
| Number of papers which had SNIP score ^d^ | 22.8±12.3 | 62.5±30.0 |
| SNIP per publication ^d^ | 1.2±0.3 | 1.2±0.3 |
| Number of GIA projects obtained ^e^ | 2.9±1.7 | - |
| Amount of GIA projects obtained (thousand yen) ^e^ | 21190.0±24477.5 | - |

^a^ Based on publication during 2012-2016 for the promising researchers and during 2007–2011 for supervisors, respectively. Obtained from SciVal; ^b^ Obtained from ISM+Neo4j that consisted of 30 years of Web of Science data on articles published until 2016. ^c^ Calculated using citation count until 2016 (ISM+Neo4j); ^d^ Calculated using citation count until 2021 (SciVal); ^e^ During 2012-2021. Obtained from the Database of Grants-in-Aid for Scientific Research (National Institute of Informatics, Japan. GIA, Grants-in-Aid. ^f^ Excluding duplicates from the 30 identified as promising researchers’ supervisors. Values are mean ± S.D.
